# Topography as a modifier of breeding habitats and concurrent vulnerability to malaria risk in the western Kenya highlands

**DOI:** 10.1186/1756-3305-4-241

**Published:** 2011-12-23

**Authors:** Harrysone E Atieli, Guofa Zhou, Ming-Chieh Lee, Eliningaya J Kweka, Yaw Afrane, Isaac Mwanzo, Andrew K Githeko, Guiyun Yan

**Affiliations:** 1 Climate and Human Health Research Unit, Centre for Global Health Research, Kenya Medical Research Institute, P.O. Box 1578-40100, Kisumu, Kenya; 2 Community Health Department, School of Public Health, Kenyatta University, P.O Box 43844-00100, Nairobi, Kenya; 3 Program in Public Health, College of Health Sciences, University of California, Irvine, CA 92697, USA; 4 Tropical Pesticide Research Institute, Division of Human and Disease Vectors Control, Mosquito Section. P.O. Box 3024, Arusha, Tanzania

## Abstract

**Background:**

Topographic parameters such as elevation, slope, aspect, and ruggedness play an important role in malaria transmission in the highland areas. They affect biological systems, such as larval habitats presence and productivity for malaria mosquitoes. This study investigated whether the distribution of local spatial malaria vectors and risk of infection with malaria parasites in the highlands is related to topography.

**Methods:**

Four villages each measuring 9 Km^2 ^lying between 1400-1700 m above sea level in the western Kenya highlands were categorized into a pair of broad and narrow valley shaped terrain sites. Larval, indoor resting adult malaria vectors and infection surveys were collected originating from the valley bottom and ending at the hilltop on both sides of the valley during the rainy and dry seasons. Data collected at a distance of ≤500 m from the main river/stream were categorized as valley bottom and those above as uphill. Larval surveys were categorized by habitat location while vectors and infections by house location.

**Results:**

Overall, broad flat bottomed valleys had a significantly higher number of anopheles larvae/dip in their habitats than in narrow valleys during both the dry (1.89 versus 0.89 larvae/dip) and the rainy season (1.66 versus 0.89 larvae/dip). Similarly, vector adult densities/house in broad valley villages were higher than those within narrow valley houses during both the dry (0.64 versus 0.40) and the rainy season (0.96 versus 0.09). Asymptomatic malaria prevalence was significantly higher in participants residing within broad than those in narrow valley villages during the dry (14.55% vs. 7.48%) and rainy (17.15% vs. 1.20%) season. Malaria infections were wide spread in broad valley villages during both the dry and rainy season, whereas over 65% of infections were clustered at the valley bottom in narrow valley villages during both seasons.

**Conclusion:**

Despite being in the highlands, local areas within low gradient topography characterized by broad valley bottoms have stable and significantly high malaria risk unlike those with steep gradient topography, which exhibit seasonal variations. Topographic parameters could therefore be considered in identification of high-risk malaria foci to help enhance surveillance or targeted control activities in regions where they are most needed.

## Background

One fifth of the African population lives in malaria epidemic prone areas (desert fringes and highlands) [1] where all age groups are at risk of clinical malaria due to the limited acquired immunity. The prevention of malaria in these vulnerable populations is one of the priorities for the governments, African leaders and international agencies [2]. In Kenya, transmission of *Plasmodium falciparum *in the highlands has been a re-emerging problem in several regions in the last three decades [3]. The malaria situation has been getting worse partly due to resistance to anti-malarial drugs and lack of sufficient vector control measures [4,5]. Furthermore, it has been demonstrated that malaria epidemics in the Western Kenya highlands are partly driven by climate variability [6,7]. The impact of malaria epidemics on human morbidity and mortality may become more severe because climate variability is predicted to become more frequent and intense [7]. Understanding the epidemiology of malaria transmission and variations that occur within areas with close proximity in the highlands would support the improvement of an area specific national strategy plan for prevention and transmission control. It is therefore, necessary to explore possible factors fuelling these changes in transmission so as to identify vulnerable villages to allow interventions to be directed at these high-risk communities [8].

Topography has long been recognized to be one of the factors associated with malaria [9,10] due to its association with cooler temperatures that slow the development of anopheline vectors and the *Plasmodium *parasites they transmit [5,11]. The topography of the highlands comprises hills, valleys and plateaus. Rivers and streams run along the valley bottoms in the valley ecosystem and swamps are a common feature. Unlike in lowland plains, where drainage is poor and mosquito breeding habitats have an extensive distribution, the majority of breeding habitats in the hilly highlands are confined to the valley bottoms because the hillside gradients provide efficient drainage [12]. Variation in the local shape of the land may also play an important role in determining regions of suitability for mosquito breeding at smaller spatial scales [13]. Depending on the variation in local valley shape, malaria risk may diminish within a few hundred meters from known breeding sites [14,15], although a number of vector and environmental factors have been found to influence this range [16,17].

As characterized by Balls *et al*. in Tanzania, the risk of malaria in the highland region is a broad altitudinal trend modified at smaller spatial scales by local topography [13]. Within the highlands, there are broader valleys with slow gradients while others are narrower with stiff gradients. The broader valleys with 'U' shaped bottoms tend to have fairly extensive distribution of breeding habitats compared to the narrow 'V' shaped valleys. Many vector control efforts assume the same strategy of malaria control. While this may be largely true in the lowlands, such an assumption is not true in the highlands. This assumption may lead to expensive and extensive control efforts. It would be advisable to categorize the villages for targeted interventions. Recognizing and understanding consistent foci of these ecological factors would therefore permit control efforts to be directed at specific geographic areas, reducing costs and increasing outcome. Using topography as a factor, this study assessed spatial patterns of mosquito larval breeding habitats, vectors and malaria incidence in four villages with variation in local valley shape in an epidemic-prone area of the Western Kenyan highlands.

## Methods

### Study site

**Figure 1:**
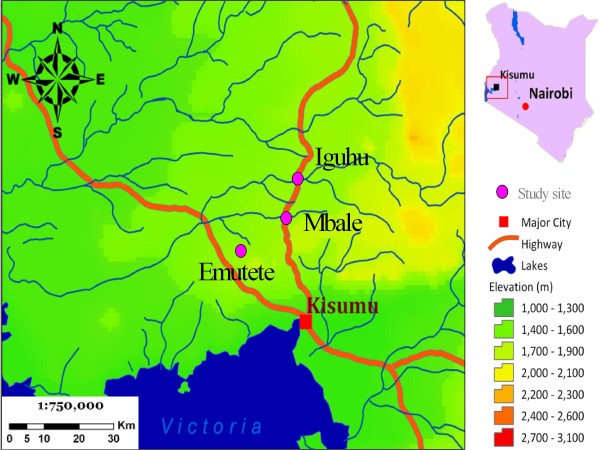
**Study area map showing three study sites**. Study area map showing three study sites: Iguhu in Kakamega district, Mbale and Emutete in Emuhaya district.

The four study villages are found in; Iguhu (0°11' N, 34°44' E, 1,420-1,570 m a.s.l) in Kakamega district, Emutete (0°02' N, 34°38' E, 1,480-1,650 m a.s.l) in Emuhaya district and Mbale (0°07' N, 34°72' E, 1,420-1,650 m a.s.l) in Vihiga district situated in the highlands of Western Kenya (Figure 1). They are located within a grid of 3 × 3 km^2 ^and densely populated with average population density of over 900 persons/km^2 ^[18]. The principal occupations of inhabitants in both villages included subsistence (maize and some vegetables) and animal husbandry (cattle, goats, sheep and chickens). The climate in western Kenya generally consists of a bimodal pattern of rainfall, with the long rainy season from April to June and a short rainy season in November and December. There is no clear dry season, but usually there is less rainfall from July to September. January and February are the hottest and the dry months. During this study the short rain season of November - December was chosen for survey. Unlike the long rainy season, during this season the rains are fairly calm and habitats tend to stabilize and stay longer to hold the aquatic life cycle of mosquitoes. During the long/heavy rainy season (April-May), most larvae habitats in steep V-shaped valleys are flushed down stream. This flush effect results in very low larvae and adult vector densities not meaningful for comparison purposes with those in the broad U-shaped valley ecosystem. The epidemiology of malaria in the study areas is defined as epidemic-prone districts (risk class 5-20%). Malaria transmission peaks during the long rainy season. As in other highland areas of East Africa, the predominant vector is *An. gambiae *s.l. (97.5%) [19]. ITNs use supplied through the universal coverage by the Government is the main vector control strategy in this area. During the period of this study, ITN ownership in all the four study sites was similar at an average of 71% with actual usage at 56% [5].

### Digital elevation model

**Figure 2:**
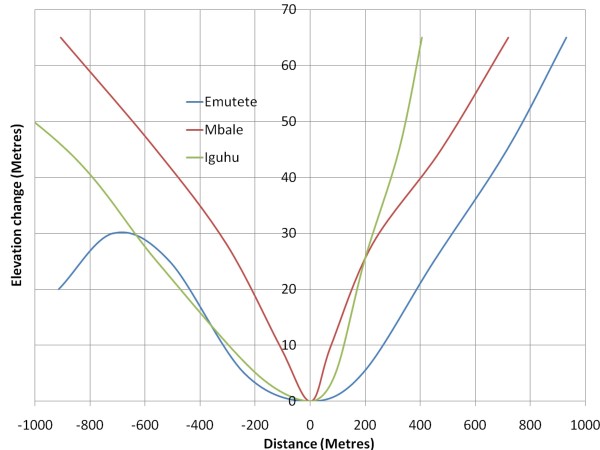
**Terrain profile for "U" and "V" shaped valleys**. Terrain profile for "U" and "V" shaped valleys as generated from actual cross section elevation values.

**Figure 3:**
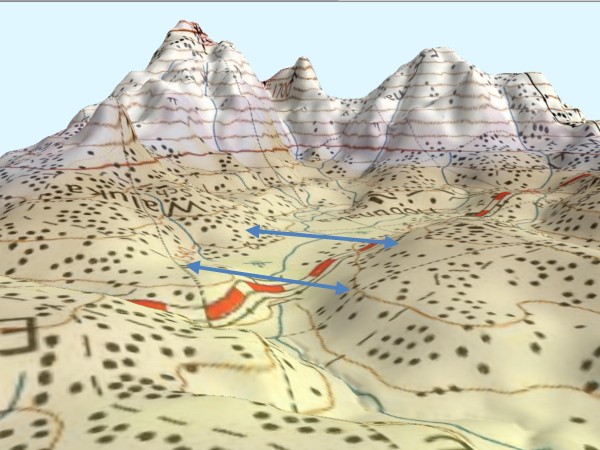
**A 3D map**. A 3D map of a broad "U"-shaped valley in Emutete.

**Figure 4:**
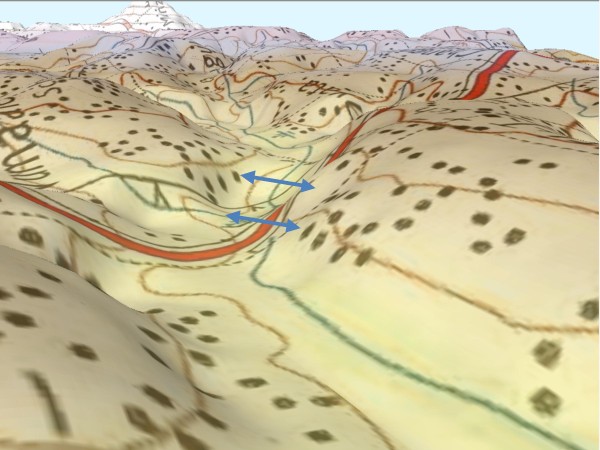
**A 3D map of a narrow "V"-shaped valley in Mbale**.

Digital files consisting of points of elevation, sampled systematically at equally spaced intervals were used in the generation of terrain profiles between selected points (Figure 2) to display the shape of the valleys. To determine how topography and terrain characteristics are associated with the availability and stability of mosquito breeding habitats and malaria transmission, 3-D images of broad (Figure 3) and narrow (Figure 4) shaped valleys were constructed using 30 meters and 1- meter horizontal and vertical resolutions. Elevation data was obtained from contour maps and satellite images of LANDSTAT and IKONOS of Western Kenya. Using these maps the contours were digitized and interpolated to create a DEM. Details of the peaks and valleys in the terrain were represented with small grid spacing. Elevation recorded by a hand-held global positioning system (GPS) receiver (e Trex HC series, Garmin International, Inc) helped to fill in points that may not be present in the contour maps. Ground truthing was carried out using GPS units of a 2 × 2 kilometer cross-section of the valleys to ascertain the accuracy of topography maps. Where there was variance, the GIS data was used as inputs in the model.

The elevation differences between upstream and downstream drainage points were determined to indicate the efficiency of drainage thus the stability of the breeding habitats. The surface area with no slope at the bottom of the valleys was determined. Valley shape is one of the terrain characteristic which is a key driver to malaria in the highlands [20]. The U-shaped valleys were defined as broad valleys with slow moving rivers or streams and have poor drainage. Their river flow slope change rate is 1% and with a flat surface from the river edge of > 10 meters. On the other hand, the V-shaped valleys have a narrow bottom with a fast flowing river or stream and have good drainage. Their river flow slope change rate is 10% and with a flat surface of < 10 meters from the river edge (Githeko *et al*. unpublished data). These parameters were compared in a pair of villages within narrow V-shaped and another pair within flat-bottomed U-shaped valleys to determine whether there is consistency in the characteristics as explained above. The difference in topographic parameters between broad and narrow valley and their association with occurrence and stability of malaria risk was determined.

### Selection of study houses and participants

Using a handheld GPS unit, coordinates of all houses within the sites were numbered and mapped. A hundred and twenty houses were randomly selected from each of the four villages making a total of 480 houses. In each village, 60 houses were assigned from both sides of the river from the valley bottom to uphill. Owners of selected houses were requested to sign a freely administered informed consent form while guardians of minors signed assent forms for participation in the study, and for entomologic, parasitological and questionnaire surveys.

### Entomological survey

Adult indoor resting mosquitoes were collected using the Pyrethrum Spray Collection (PSC) method [21] during the dry (February-March 2010, low transmission season) and the rainy (November-December, 2010, high transmission season) seasons from all the study houses (120 houses from each village in each season). Likewise, larval distribution surveys were done during the same period concurrently. A field team comprised of a researcher, technician and field assistant who surveyed the entire village for all aquatic habitats. Twenty dips were made in each habitat using a standard dipper (350 ml) manufactured by Bid Quip Products, Inc. California, USA. Small habitats were dipped as many times as possible. Larval densities were then adjusted to larvae per dip. Aquatic habitats with or without anopheline presence were identified, recorded and characterized into habitat type. Similarly, using handheld GPS, these habitats were mapped. Samples were taken to the Kenya Medical Research Institute (KEMRI) laboratory in Kisumu, for counting and morphological identification to species, and adult mosquitoes were classified according to their gonotrophic stages.

### Malaria infection survey

Occupants from study houses sprayed each season for vector collection, (who freely consented to participate in the study), were screened for malaria parasites during both the dry and the rainy season. Thin and thick blood smears were taken in the field and the slides were stained with 4% Giemsa for 30 minutes [22]. Households with absentees were revisited the following day to recruit those missing at the first visit. Symptomatic participants with positive slide tests were offered free treatment with artemisinin-based combination therapy (ACT) at the nearby health facility according to Kenya national MOH guidelines [23]. Participants with complicated malaria cases during our survey were advised to visit the nearest health facility and transportation was provided for those who needed help to get to the facility.

### Statistical analysis

Data was collected and entered in Excel spread sheets (Microsoft Corporation) and statistical analysis was performed by the use of STATA SE 9 (StataCorp LP, 4905 Lake Way Drive, College Station, TX 77845 USA). Study results were categorized into two, those closest (≤ 500 m) to the main river line valley where the majority of breeding habitats occurred at the valley bottom (VB) while those above that distance of 500 m were considered to be uphill (UH). A distance of 500 m was chosen having been used successfully elsewhere [10], although published distance of risk gradients vary [24] and sharp declines in risk are generally reported at greater distances [25]. For comparison, density/house of indoor collected vectors, positive larval occurrence and abundance and the rate of malaria within the valley bottom and uphill between broad and narrow shaped valley villages were calculated.

During analysis, data from the two broad valley villages were grouped together, likewise those from the two narrow valley villages were grouped together after preliminary results confirmed that in addition to their topographic aspects similarity, there were no intra-specific differences in both adult vectors and larval abundance and distribution characteristics. To determine whether there were differences in the abundance of adult vectors between the valley bottom and uphill in each particular village, i.e. broad and narrow shaped valley villages, density of vectors/house in houses located at the valley bottom were compared by t-test to those located uphill during both the rainy and the dry season. Similar comparisons using chi-square test were done to determine the difference in occurrence of positive larval habitats between areas located at the valley bottom and uphill during the dry and the rainy seasons in the broad and narrow shaped valley villages. Chi-square test was carried out to examine whether patterns of malaria surrounding households closer to the valley bottom locations might appear analogous to those uphill in broad and narrow shaped valley villages during both the rainy and dry seasons. Inter valley shape comparison between broad and narrow shaped villages were done comparing adults, larval and malaria occurrences. As for the abundance and distribution of adult vectors, a t-test was used, whilst a chi-square test was used for both positive larval habitats and malaria cases. Multivariate analysis- Tukey HSD test was done to determine the most predictive independent variable among valley shape, altitude and season for the occurrence of larvae, adult vectors and malaria cases as dependent variables.

### Ethical consideration

Ethical clearance was obtained from the Ethical Review Committee of Kenya Medical Research Institute, "Ecology of African highland malaria (II), SSC No. 1382 (N)" dated May 15^th ^2008 and the Institutional Review Board of the University of California at Irvine. A freely administered informed consent with interpreters was given to residents for participation in the study.

## Results

### Larval habitats survey

**Figure 5:**
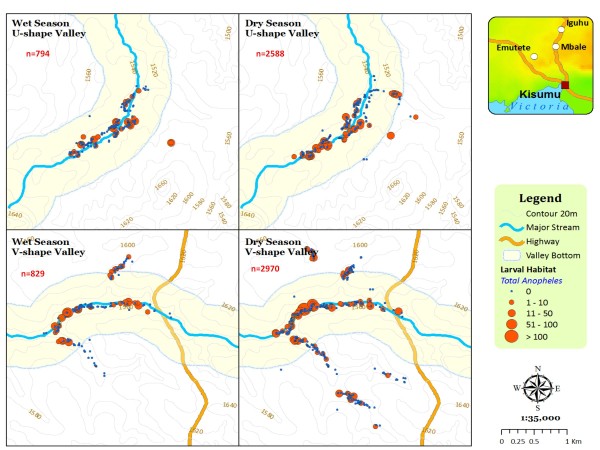
**The distribution and abundance of seasonal malaria vector larvae positive habitats**.

Overall, the broad U-shaped valleys had higher vector densities and malaria infections than the narrow/steep V-shaped valleys both during the dry and rainy seasons (Table 1). The density of both *An. gambiae s.l *and *An. funestus *per dip found in aquatic habitats in broad U-shaped valleys were significantly higher than those found in the narrow V-shaped valleys in both the rainy and the dry seasons. During the rainy season survey, 655 aquatic habitats (total negative and positive) were identified in the study areas. Of these habitats, 376 (57.40%) were found in the broad U-shaped valley sites. During the dry season, a total of 1,338 habitats were found in the study sites and of these, 574 (42.90%) were in the broad U-shaped valley site. Of the 376 aquatic habitats found in the broad U-shaped valley area during the rainy season, 97 (25.90%) were positive for anopheline larvae while of the 574 habitats found during the dry season, 140 (24.39%) were positive for anopheline larvae. In the narrow V-shaped valley, of the 279 habitats found during the rainy season, 78 (27.95%) had anopheline larvae while during the dry season 108 (14.13%) habitats out of 764 habitats were positive of anophelines. Table 2 shows the percentage of habitats positive with anopheline larvae categorized by habitat location of valley bottom (≤500 m from the valley river line) and uphill area (≥500 m from the valley river line). During univariate analysis, anopheline larvae occurrence was associated with many topographic variables. Altitude was negatively associated with occurrence of anopheline larvae (t = -2.81, P < 0.005). Habitats positive for anopheline larvae were evident in lower altitude areas along the main river bank, 500 m distance both sides of the river, categorized as valley bottom (Figure 5). Valley shape, either U or V-shape (t = 3.77, P < 0.0001) and wet season (t = 3.97, P < 0.0001) were positively associated with anopheline occurrence with more anopheline positive habitats in broad than narrow valleys. Likewise, during rainy season, more positive habitants were identified than during the dry season. Multivariate analysis showed that, *An. gambiae s.l *larvae positive habitat occurrence was significantly dependent on the valley shape of either broad U-shaped or narrow V-shaped valley irrespective of the season. The broad U-shaped valley had a significantly higher probability of positive larval habitat occurrence than the narrow V-shaped valleys (P = 0.024, Tukey HSD test) during both the rainy and the dry season. In contrast, although there were more *An. funestus *positive habitats in the broad valley than the narrow valley, their occurrence was strongly season dependent (P = 0.06, Tukey HSD test).

**Table 1:** Summary of vector densities and parasite prevalence by valley shape and season

Valley shape	U-shaped valleys		V-shaped valleys	
**Season**	**Rainy**	**Dry**	**Rainy**	**Dry**

Larvae density/dip				
*An. gambiae s.l *	1.66	1.89	1.19	0.89
*An. funestus *	2.19	4.39	2.54	4.11
Adults vectors density/house				

*An. gambiae s.l *	0.77	0.46	0.08	0.38
*An. funestus *	0.19	0.17	0.01	0.02

Parasite prevalence (%)	17.15	14.55	1.20	7.48

**Table 2:** Percentage of habitats with malaria vector larvae within valley bottom and uphill locations in different valley shapes and different seasons

	Rainy season (2009)		Dry season (2010)	
		
Valley shape	Valley bottom	Uphill	Valley bottom	Uphill
U-shape	25.6	26.8	25.5a	21.5a
V-shape	28.7A	26.7A	15.9Bb	12.6Bb

χ^2^-test at significant level of 5%. Different capital means; seasonal difference at same location **within valley shape**. i.e. a difference at the valley bottom between rainy and dry season in V-shape valleys. Different small letters; means difference within location **between valley shapes **in each season. i.e. a difference between U and V-shape at both valley bottom and uphill during the dry season.

### Adult malaria vector survey

**Figure 6:**
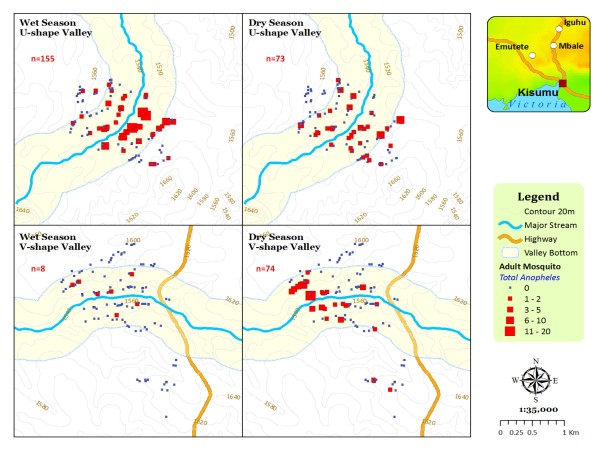
**The distribution and abundance of seasonal indoor malaria vectors in different valley shapes**.

Of the total indoor resting adult mosquitoes collected, 54.75% and 90.64% were found in the houses located in the broad valley during the dry and rainy seasons respectively. Houses in the broad valley ecosystem had significantly higher densities (P < 0.05) of vectors than houses in the narrow valley ecosystem during the dry and rainy season (Figure 6). During the dry season, of the total houses sampled in the broad valley, 38.33% were positive for at least one vector while during the rainy season 41.89% were positive. In the narrow valley, 16.25% of the houses were positive for malaria vectors during the dry season while 6.11% were positive in this valley during the rainy season. Adult vector densities were higher in houses in the broad rather than in the narrow valley ecosystem during both the dry (0.64 versus 0.33) and rainy (0.80 versus 0.08) seasons. When categorized by valley location, both the valley bottom and uphill location houses of the broad valley had significantly higher densities of vectors when compared to similar locations in the narrow valley (Table 3). In univariate analysis, *An. gambiae s l*. densities were positively associated with valley shape (t = 5.70, P < 0.0001) with more vectors found in houses within the broader U-shaped valley (Figure 6). Altitude was negatively associated with indoor vector densities (t = -4.91, P < 0.0001). There was no association of vector densities with season. Two models were generated during multivariate analysis, including valley and altitude variables. In the first model, where indoor vector densities and only valley shape was used as an independent variable, valley shape improved the prediction. Despite being negatively associated with indoor vector densities, addition of altitude in the second model did not improve the model prediction for presence of indoor vectors. The additional proportion variability accountable for by altitude was 0.9%, an evident lack of significance impact by addition of altitude in the prediction model.

**Table 3:** Percentage of houses with malaria vectors within valley bottom and uphill in different valley shapes and seasons

	Rainy (2009)		Dry season (2010)	
		
Valley shape	Valley bottom	Uphill	Valley bottom	Uphill
U-shape	43.9a	13.9Aa	46.0a	27.7Ba
V-shape	8.0Ab	2.9b	24.8Bb	4.9b

### Malaria infection rates

**Figure 7:**
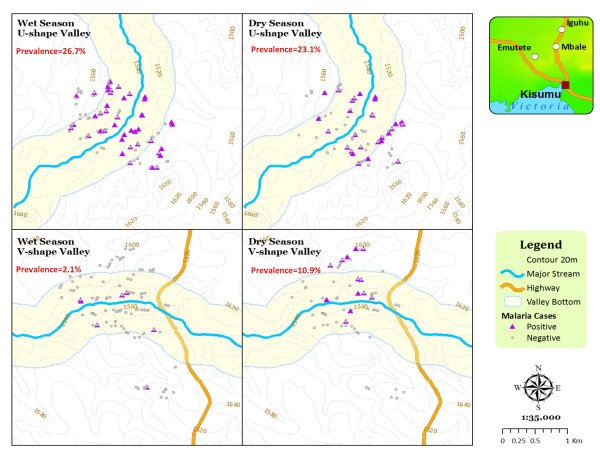
**Seasonal distribution of microscopy malaria positive**. Seasonal distribution of microscopy malaria positive participants in two different valley shapes.

Risk of malaria infection differed across the valley shapes (Table 4). Malaria prevalence was significantly higher in participants residing within broad valley villages than those in narrow valley villages during the dry (14.55% vs. 7.48%) and rainy (17.15% vs. 1.20%) seasons (Figure 7). In univariate analysis, malaria infections were associated with topographic variables. Malaria infections were positively associated with valley shapes while season and altitude had a negative association. Broad valley shape was associated with high malaria infections (t = 9.96, P < 0.0001). Both season and altitude had a negative association with malaria infection occurrence, t = -2.49, P < 0.013 and t = -5.83, P < 0.0001 respectively. High rainy season was likely to reduce infections probably because of larval wash effect leading to low vector production. High altitude was likely to be associated with fewer malaria cases. Multivariate models using topographic independent variables of valley shape, altitude and season all predicted malaria infection cases. In every model repetition, adding valley shape and altitude improved prediction of malaria infection cases. However, valley shape predicted malaria infection cases better than altitude in all the repetitions. Valley shape and altitude generated the best prediction model with the highest coefficient.

**Table 4:** Malaria parasite positive rates in participants within valley bottom and uphill in different valley shapes and different seasons

	Rainy (2009)		Dry season (2010)	
		
Valley Shape	Valley bottom	Uphill	Valley bottom	Uphill
U-shape	20.6a	12.7a	15.2a	13.9
V-shape	1.6Ab	0.7Ab	7.8Bb	7.8B

## Discussion

Malaria risk mapping requires inclusion of factors related to vector distribution, human-vector contact, human practices, and the environmental context in which they occur [26]. Highland topographic features restrict the spatial distribution of vector breeding habitats confining them to the valley bottom [12,27]. Furthermore, it restricts high intensity of exposure to malaria at the valley bottom resulting in a heterogeneous incidence of morbidity when compared to hilltop. Identification of area specific topographic features has important implications in classification of malaria epidemiology for specific micro-regions. Moreover, accurate prediction of malaria vector occurrence and plasmodium transmission risk is essential in heterogeneous environments to permit focal cost effective intervention strategies and heightened surveillance in the regions that require them the most [20]. Results of this investigation concur with previous studies demonstrating associations between the occurrence of malaria vectors and malaria risk and topographic characteristics, specifically the shape of the valley in the highlands where malaria epidemics occurs. Importantly, however, it demonstrates that factors associated with malaria are not necessarily predictive of it, most likely because strong correlations between environmental factors can lead to confounded relationships.

In highland regions of East Africa, where unstable malaria transmission may result in part from the very low numbers of anopheline mosquito vectors [28], the proximity of houses to locations with suitable topography for mosquito breeding may be an important determinant of malaria risk [24]. During this study, spatial surveys of the availability of larval habitats and presence of larvae in these habitats showed that, the actual numbers of positive larval habitats in broad valley regions were greater and stable in both the rainy and the dry seasons. In contrast, percentages of positive larval habitats were season dependent in narrow V-shaped valleys with significantly high occurrence during the rainy rather than during the dry season. This can be explained by the fact that unlike the broad U-shaped valleys that are characterized by meandering slow moving rivers, poor drainage and with large surfaces to hold water at the valley bottom suitable for larval breeding, the narrow V-shaped valley systems are characterized by fast running rivers at the valley bottoms. They too have steep slopes that provide good drainage in the area and so there are few vector breeding habitats in these ecosystems. Similarly, during the adult vector survey, households within the broad U-shaped valley had higher densities of vectors per house during both the dry and rainy seasons compared with those within the narrow V-shaped valley. This result concurs with earlier studies in the highlands which indicated that anopheline larval habitats and malaria transmission were generally clustered near the streams and rivers with poor drainage [12,26]. Broad U-shaped valley regions were therefore suitable for larval breeding and productivity of adult vectors than the steep narrow V-shaped valley regions.

High larval and adult vector abundance within broad rather than narrow valleys further explains why spatial variation in malaria transmission in the highlands is a function of the terrain characteristics [25]. This phenomenon also sheds more light on the reason why there is heterogeneity in malaria transmission in the highlands [29] and with variations in close proximity areas within the same region. Steep V-shaped valleys experience fast surface water and river flows during the rains. These events do not allow formation of habitats that would stay long enough to sustain survival of the mosquito aquatic life cycle. These ecosystems therefore experience wash effect on potential larval habitats. Habitats with either eggs or newly hatched larvae are often washed down-stream with running water. This fact was evident where vector densities in a narrow valley shape exhibited significant seasonality in distribution while those in broad valleys had similar distribution across the seasons (Figure 6). Probably because of the wash effect, the narrow valley exhibited unusual vector densities during the rainy season. Unlike in the broad valley where there were fairly higher densities of vectors/house during the rainy season, the narrow valley had significantly fewer vectors during the rainy than the dry season. Vector abundance and distribution were associated with valley shape, location and season. Similar studies on vector distribution in this region have shown that low-lying flat areas and reclaimed swamps are highly productive than steep terrains habitats [20,27,29,30]. These findings using topographic parameters to identify area specific larvae and adult vectors spatial distribution can be utilized to characterize risk regions with close proximity for the purpose of targeted larval or adult vector control. Unlike blanket control, a targeted approach would use limited resources with expected greater outcomes.

The magnitude of the differences in vector abundance (2-10-fold difference) and 2-14-fold difference in malaria incidences between the narrow and broad valley regions during the dry and the rainy season respectively was striking, suggesting that even within this small area, risk of malaria ranges from very low to quite high depending on the shape of the valley. The ecological factors independently associated with increased malaria risk in this study - a broad U- shape valley, lower altitude and rainy season - are all physical contributors to increased malaria risk in the highlands [10,20]. Moreover, the associations of these risk factors with vector abundance and malaria incidence were strong, consistent, similar over the seasons, and highly significant. The results of this study indicate that as a topographic factor, the shape of the valley either broad U-shaped or narrow V-shaped and distance from the valley bottom predicted the presence of both anopheline positive aquatic habitats and indoor malaria vectors and thus are highly predictive of malaria patterns in this small region. People living in areas within the broad valley shape appeared to be at significantly greater risk of fairly stable malaria infections than those living in areas of narrow valley shape. The non-homogeneous distribution of larval breeding habitats and adult vector spatial distribution between these two valley shape ecosystems may, consequently, lead to focal malaria transmission and heterogeneous human exposure to malaria. It can thus be expected that the malaria transmission profile in the highlands is influenced not only by hydrological factors, but also by topographic factors and distance from the foci of transmission. This heterogeneity in transmission can lead to variable stability of malaria transmission in space with some areas having stable and others unstable transmission. This would lead to different sensitivities to epidemics within relatively short distances in the highlands. Future studies should assess the utility of valley shape as a topographic factor for malaria risk prediction across larger and more geographically diverse areas, especially at scales useful for National division of malaria control to target interventions. Such replication of this work will be required before conclusions can be drawn about the utility of these methods elsewhere.

## Conclusions

Given the cost of malaria intervention, accuracy of predicting and classifying high-risk foci in unstable malaria transmission regions is crucial. These findings indicate that malaria control programs operating in similar rugged terrain and highland regions might use topographic and local geographic variance to efficiently identify and derive risk maps of locations that are highly suitable for transmission and which may benefit from targeted interventions and enhanced vigilance.

## Competing interests

The authors declare that they have no competing interests.

## Authors' contributions

HA carried out the field surveys, assembled data, analyzed and drafted the manuscript. GZ and MCL performed statistical analysis and revised the manuscript. EJK, YA and IM participated in study coordination and revised the manuscript. AG and GY designed the study and helped with manuscript preparation. All authors read and approved the final manuscript.
